# New Insights Into the Development of the Anterior Abdominal Wall

**DOI:** 10.3389/fsurg.2022.863679

**Published:** 2022-03-31

**Authors:** Jose Bouzada, Carolina Gemmell, Marko Konschake, R. S. Tubbs, Elisabeth Pechriggl, Jose Sañudo

**Affiliations:** ^1^Department of Anatomy and Embryology, University Complutense of Madrid, Madrid, Spain; ^2^Department of Anatomy, Histology and Embryology, Institute of Clinical and Functional Anatomy, Medical University of Innsbruck, Innsbruck, Austria; ^3^Department of Neurosurgery, Tulane Center for Clinical Neurosciences, Tulane University School of Medicine, New Orleans, LA, United States; ^4^Department of Neurology, Tulane Center for Clinical Neurosciences, Tulane University School of Medicine, New Orleans, LA, United States; ^5^Department of Anatomical Sciences, St. George's University, St. George's, West Indies; ^6^Department of Structural and Cellular Biology, Tulane University School of Medicine, New Orleans, LA, United States; ^7^Department of Surgery, Tulane University School of Medicine, New Orleans, LA, United States; ^8^Department of Neurosurgery, Ochsner Neuroscience Institute, Ochsner Health System, New Orleans, LA, United States; ^9^University of Queensland, Brisbane, QLD, Australia

**Keywords:** external abdominal muscle, internal abdominal muscle, transversus abdominis muscle, rectus abdominis, inguinal canal, umbilical cord, umbilicus

## Abstract

**Purpose:**

Among the few studies that have examined the development of the anterior abdominal wall, several are based on incomplete “series”, substituted in many cases by non-human specimens.

**Material and Methods:**

In total, 19 human embryos corresponding to Carnegie stages 15–23, 36 fetuses with estimated gestational ages ranging from 9 weeks to term, and eight neonates were included in this study. All specimens belong to the collection of the Department of Anatomy and Embryology at the Complutense University of Madrid.

**Results:**

The muscles of the anterior abdominal wall appear in the dorsal region at stages 15 and 16 (33–37 days). At stages 17 and 18 (41–44 days), this muscular mass grows ventrally and splits into two sheets: the external abdominal oblique muscle and the common mass of the internal abdominal oblique, and the transversus abdominis muscles, all of which end ventrally in the primitive condensation of the rectus abdominis. In embryos at stages 19 and 20 (48 days), the anterior abdominal wall continues to show an umbilical hernia in the amniotic cavity. However, a narrow neck is apparent for the first time and there is a wider anterior abdominal wall below the hernia made up of dense mesenchyme tissue without layers and showing the primordia of the umbilical canal. In embryos at stages 21, 22, and 23 (51–57 days), the abdominal muscles and aponeuroses cross the midline (linea alba) covering the rectus abdominis and pyramidalis muscles while the umbilical hernia has shrunk. In fetuses during the 9th and 10th weeks, the umbilical hernia becomes encircled by the rectus abdominis muscle, its aponeurosis, and the three layers of lateral abdominal muscles, which are more developed and covered by Camper's and Scarpa's fasciae. The inguinal canal has a course and relationships like those described in adults, with Hesselbach's ligament.

## Introduction

In a previous article, the present authors described the embryonic development of the gonads and their descent involving the gubernaculum testis (1). This article considers the development of the abdominal muscles in relation to the umbilical hernia and inguinal region. There have been few studies of the embryonic development of the abdominal wall, hernia, and muscles. According to the classical studies of this topic, the core muscles grow ventrally from the paraxial mesoderm (somite) toward the medial line (2–4). The muscular column ends ventrally in straight muscles that continue dorsally as a lateral lamina, in which two sheets, internal and external, become differentiated. These form the outline of the future abdominal external oblique muscle (external sheet) and the abdominal internal oblique and transversus abdominis muscles (internal sheet) (3, 4). These classical authors considered that the muscles only become fused at the medial line at a very late stage, in 3-month-old fetuses (60 mm) (3), long after the intestine coils have returned into the abdominal cavity, which happens between the 9th and 10th weeks (2, 4–8). None of the abovementioned authors considered the growth and crossover of the abdominal muscles with their aponeuroses as one cause of the return of the intestine coils. They considered the enlargement of the umbilical arteries (2), shrinkage of the liver, and pressure of the amniotic liquid (2, 5). Only one study mentions that in two cases of omphalocele, the musculature did not reach the midline (9).

Apart from the umbilical region, the inguinal canal is most often the weakest point of the abdominal wall, and here, so-called inguinal hernias are formed (10, 11). There are other weak points where hernias could also be formed, possibly endangering the patient's life, such as the Grynfeltt's triangle, the Petit's triangle, the umbilical region, and the linea alba (12).

From an anatomical perspective, the inguinal canal is defined as the space in the abdominal wall through which the ductus deferens (in men) and the round ligament (in women) pass (12). It has two orifices, the preperitoneal or internal and the subcutaneous or external, and four walls: ventral, dorsal, cranial, and caudal (12).

Those few publications that we have found concerning the development of the abdominal wall and inguinal canal only mention a few details (13–16), or describe the development of the abdominal wall musculature without giving specific information about the inguinal region. Some assert that the inguinal canal is formed right before the end of the embryonic period during the eighth week (16), while others claim that it is formed later in the fetal period, during the 20th week of development.

The only references we found concerning the morphogenesis of the inguinal canal consider that this development occurs around a preformed gubernaculum testis, initially developing with the external or subcutaneous orifice (13, 14). Some papers mention that during the development of the inguinal canal, the gubernaculum testis is covered by the vaginal process, and this is important in the configuration of the inguinal canal (13–16).

We undertook the present study using a reliable sample of human embryonic and fetal specimens in order to increase knowledge of the development of the abdominal wall muscles and of the umbilical hernia and inguinal canal.

## Materials and Methods

In total, 19 human embryos corresponding to Carnegie stages 15–23, 36 fetuses with estimated gestational ages ranging from 9 weeks to term, and eight neonates were included in this study. All the specimens belong to the collection of the Department of Human Anatomy and Embryology at the Complutense University of Madrid. All embryos were donated to the Anatomy Department with parental consent and in accordance with Spanish Law.

The embryos were classified using the criteria proposed by O'Rahilly and Müller (17), and the fetuses were classified following the criteria described by Patten (17–19).

All the embryos, along with 25 early fetuses (weeks 9–19), were fixed in 10% neutral formalin, processed for paraffin wax histology, and serially sectioned at 10-μm thickness. The sections were then stained with hematoxylin-eosin, trichromic VOF (light green, orange G, and acid fuchsin), Bielchowsky silver, or Azan and examined under light microscopy using a Nikon Eclipse E800 microscope with Jenoptik ProGres and JVC digital cameras.

## Results

Different topographical portions of the abdominal wall, umbilical hernia, and inguinal canal appear at various stages during the embryonic period proper, and the wall undergoes posterior enlargement and maturation during the early fetal period.

### Embryos of Stages 15 and 16 (33–37 Days)

The two specimens in the 15th and 16th stages show similar development of the region of interest, so they are described together to avoid repetition.

**Figure 1:**
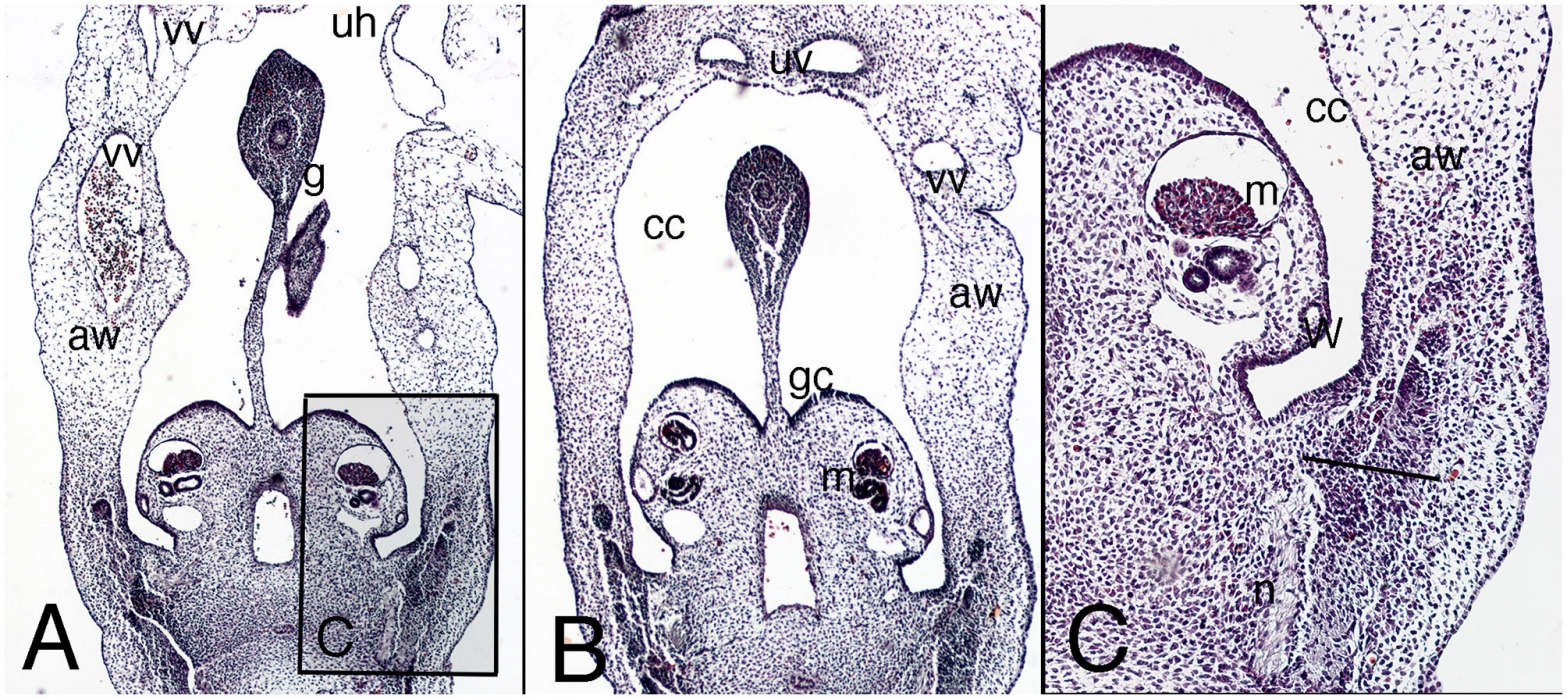
Cross section of the embryo DD-10 (stage 16). Hematoxylin and eosin staining. It represents the presumptive period of the abdominal wall. **(A)** (4X), level of the physiological hernia; the inbox **(C)** delineates the outgrowth region of the myoblast in the dorsolateral abdominal wall. **(B)** (4X), the abdominal wall just below the level of the physiological umbilical hernia with the two umbilical arteries. aw, abdominal wall; mesonephric ridge (m) behind the mesonephric duct (W); cc, celomatic cavity; g, gut; gc, gonad crest; n, nerve; uh, physiological hernia; uv, umbilical arteries; vv, vitelline vessels.

The abdominal wall contains a physiological umbilical hernia (Figure 1A). Its musculature appears as a dorsal condensation of the mesenchyme or a common myoblast mass at the dorsal level, which makes it impossible to individualize distinct muscle layers, and continues ventrally as loose mesenchyme tissue (Figures 1A,C). In the lateral abdominal wall are the vitelline veins. At the midline, the umbilical arteries, located just below the umbilical hernia, are surrounded by loose mesenchyme tissue (Figures 1A,B).

### Embryos of Stages 17 and 18 (41–44 Days)

**Figure 2:**
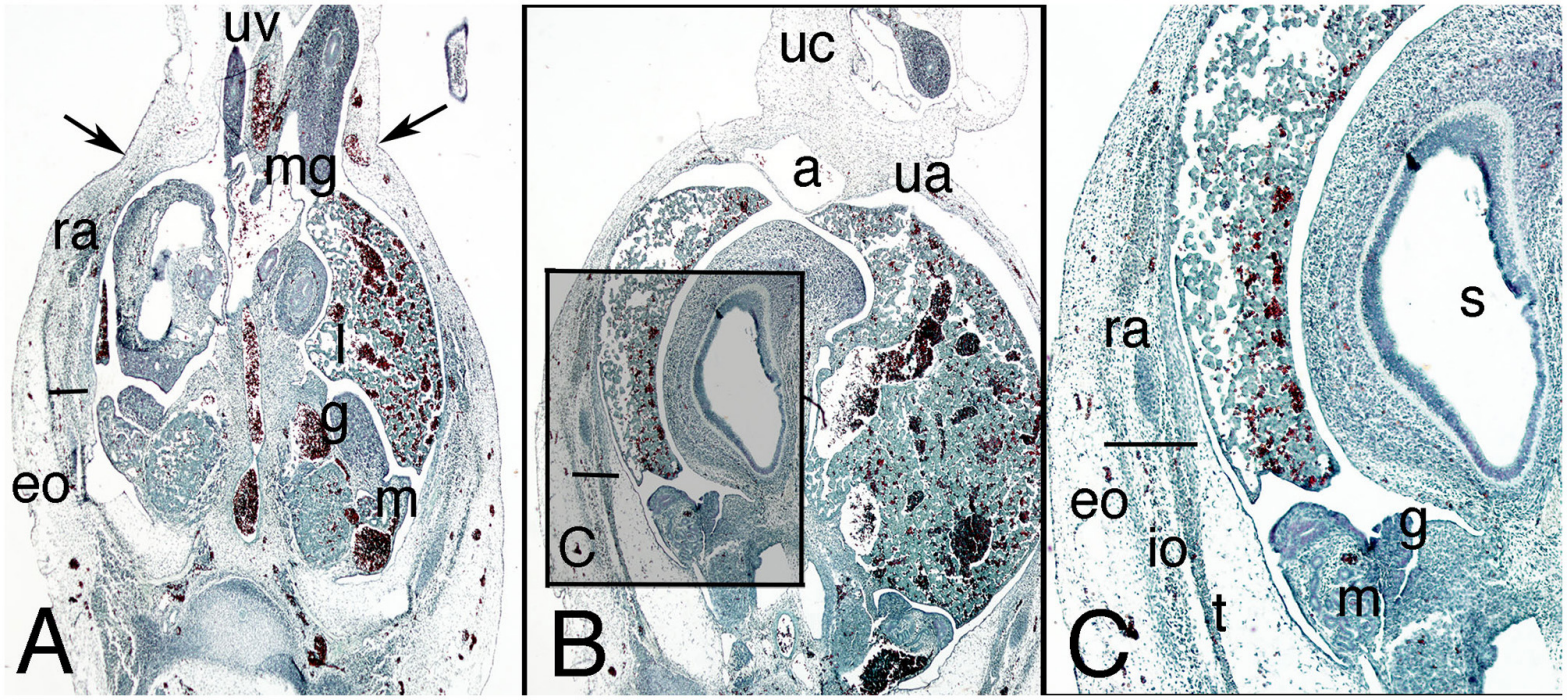
Cross section of embryo C-7 (stage 18) at abdominal level. Hematoxylin and eosin staining. **(A)** (2X) umbilical physiological hernia level; **(B)** (2X) infra-umbilical level; **(C)** (4X) box of **(B)**, showing detail of the left abdominal wall. a, alantois; eo, external oblique muscle; g, gonad; h, middle gut; io, internal oblique muscle; l, liver; m, mesonephros; mg, middle gut, ra, rectus abdominis; s, stomach; t, transversus abdominis muscle; ua, umbilical artery; uc, umbilical cord; uv, umbilical vessels. Bar, common mass of the internal and transversus abdominis muscles; arrows, neck of the umbilical hernia.

The physiological umbilical hernia persists in the abdominal wall, occupying a large portion of it, and is surrounded by the amniotic cavity (Figure 2A). Below the umbilical hernia, the abdominal wall is closed by loose mesenchyme tissue down to the pelvic brim (Figure 2B). The allantois, flanked by the umbilical arteries, appears in the dorsal abdominal wall covered by the peritoneum (Figure 2B).

In contrast to the previous stages, the musculature is differentiated along cranial–caudal and dorsal–ventral gradients, although the three studied specimens differed somewhat. In one of them, the triple-layered segmentation is not yet visible (BI-12, a more mature embryo), and in the others, the muscles appear as three independent layers, but none reaches the lateral aspect of the wall (Figures 2B,C). The external oblique muscle is clearly outlined; the other two muscles appear as a common mass that is difficult to differentiate into two layers (Figure 2C). Both sheets, the external oblique and the common mass of the transversus abdominis and internal oblique muscles terminate ventrally in a mass that represents an outline of the anterior rectus abdominis muscle (Figure 2C). The internal oblique and transversus abdominis muscles can be partially separated in their most caudal segments, because between them is the nerve stratum containing the iliohypogastric and ilioinguinal nerves, which are of extremely thick caliber.

### Embryos of Stage 19 (48 Days)

**Figure 3:**
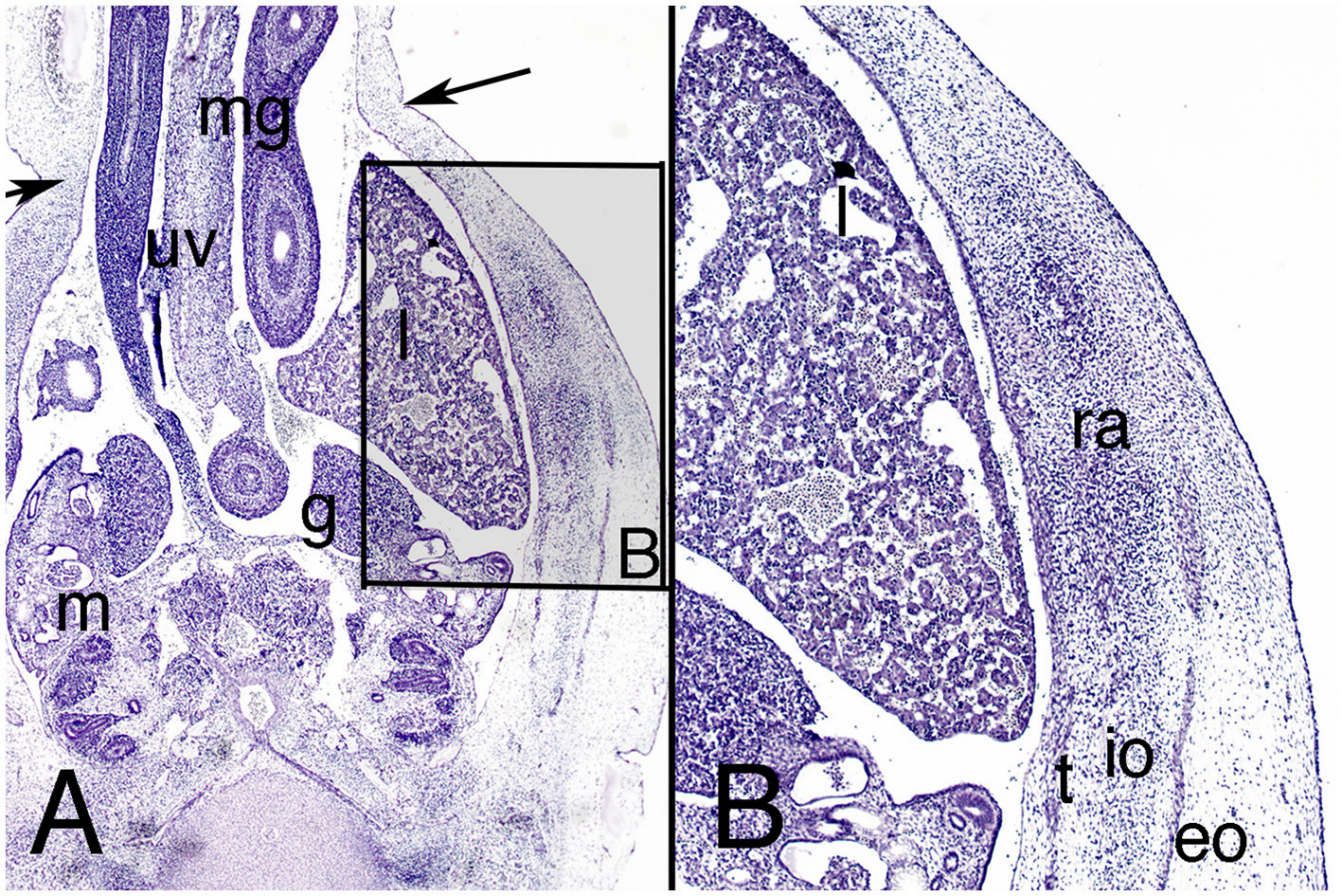
Cross section of embryo ES-18 (stage 19) at abdominal level. Hematoxylin and eosin staining. **(A)** (2X) level of the physiological umbilical hernia and **(B)** (4X) detail of the muscles of the right abdominal wall. eo, external oblique, io, internal oblique; g, gonad; l, liver; m, mesonephros; mg, midgut; ra, rectus abdominis; t, transversus abdominis; uv, umbilical vein. Arrows, neck of the umbilical hernia.

**Figure 4:**
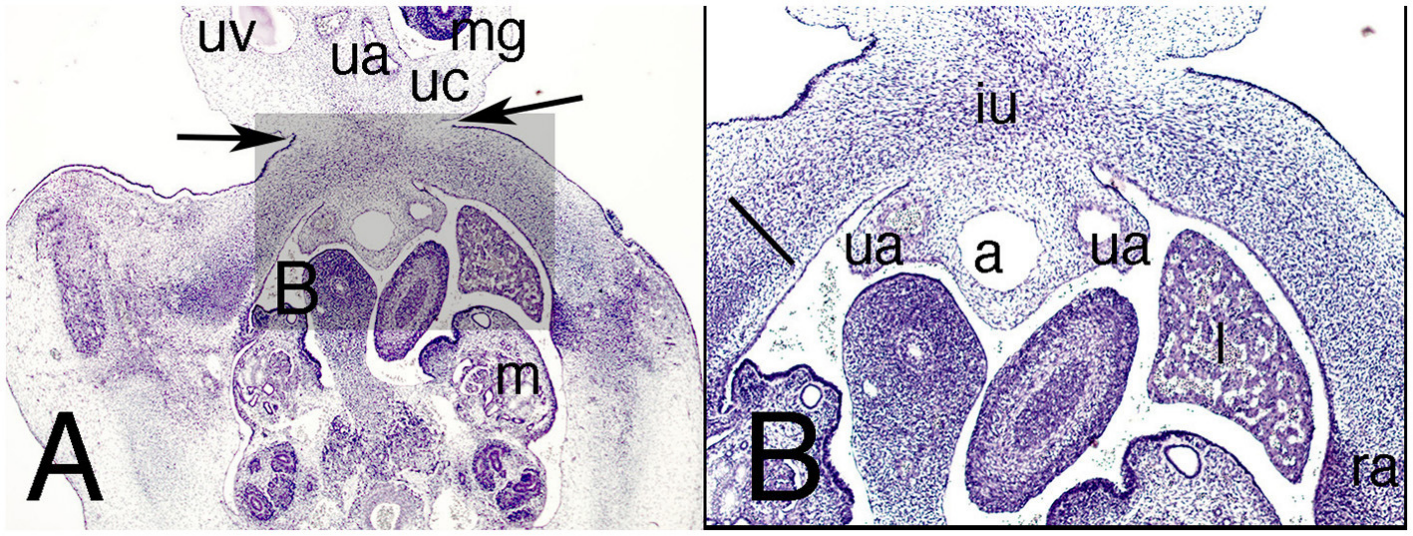
Cross section of the abdomen of the embryo ES-18 (stage 19) at infra-umbilical level. Hematoxylin and eosin staining. **(A)** (2X) level just below the physiological umbilical hernia and **(B)** (4X) at the infra-umbilical region. a, allantois; iu, infra-umbilical region; l, liver; m, mesonephros; mg, midgut; ra, rectus abdominis; ua, umbilical artery; uc, umbilical cord; uv, umbilical vein. Arrows, neck of the physiological hernia; Bar, common mass of the internal and transversus abdominis muscles in the anterior region of the abdominal wall.

The abdominal wall continues to show the physiological umbilical hernia in the amniotic cavity, but for the first time its neck starts narrowing (Figure 3A). The anterior abdominal wall below the hernia (infra-umbilical region) now consists of dense mesenchyme tissue without layers (Figures 4A,B).

However, the three lateral abdominal muscles appear to be outlined in the lateral abdominal wall for the first time (Figure 3B). The external oblique muscle is the best differentiated, but the internal oblique and transversus abdominis muscles are also differentiated, and anterior to them is the anterior abdominal rectus muscle (Figure 3B).

The allantois and umbilical arteries appear pedunculated from the dorsal surface of the ventral abdominal wall (Figure 4B).

### Embryos of Stage 20 (51 Days)

**Figure 5:**
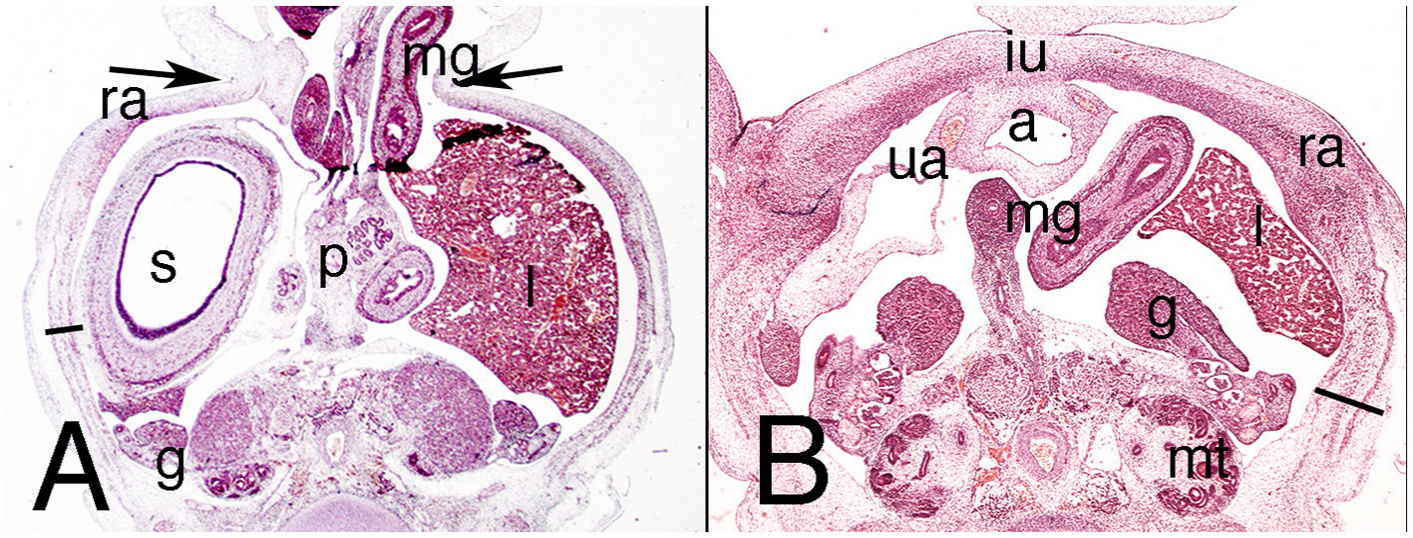
Cross section of embryo ES-20 (stage 20) at the abdominal region. Hematoxylin and eosin staining. **(A)** (1X) lower level of the umbilical hernia and **(B)** (2X) section at the infra-umbilical region. a, allantois; iu, infra-umbilical region; g, gonad; l, liver; m, mesonephros; mg, middle gut; mt, metanephros; p, pancreas; ra, rectus abdominis; s, stomach; ua, umbilical artery. Arrows, neck of the umbilical hernia; bar, differentiated lateral abdominal muscles.

The configuration of the abdominal wall is basically similar to that of the adult; it continues to show a physiological hernia at the umbilical level, but with a smaller diameter than the previous one (Figure 5A). The infra-umbilical region of the anterior abdominal wall appears wider (Figure 5B). In the umbilical region, the three lateral abdominal muscles are defined; however, the anterior abdominal muscle is less defined than the others (Figure 5A). The external oblique muscle outlines the subcutaneous orifice of the inguinal canal in the infra-umbilical region, with the gubernaculum testis inside the canal (Figure 5B).

The ilioinguinal and iliohypogastric nerves are clearly visible in the neurovascular stratum.

### Embryos of Stages 21, 22, and 23 (51–57 Days)

**Figure 6:**
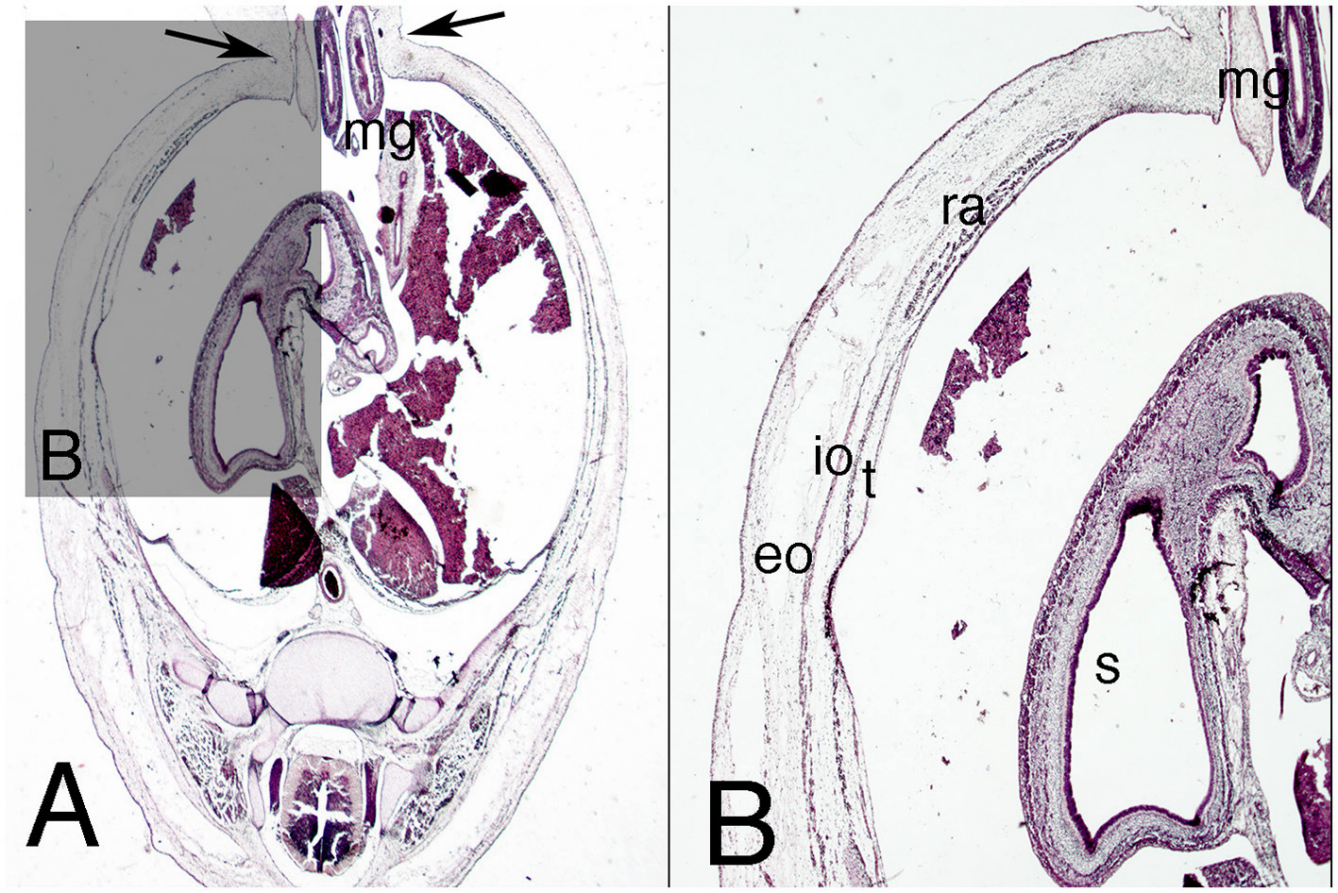
Cross sections of the abdominal cavity of the embryo MARC-1 (stage 21). Hematoxylin and eosin staining. **(A)** (1X) at the level of the umbilical hernia; **(B)** (2X) detail of muscles of the abdominal wall. eo, external oblique; io, internal oblique; s, stomach; mg, middle gut; ra, rectus abdominis; t, transversus abdominis. Arrows, neck of umbilical hernia.

**Figure 7:**
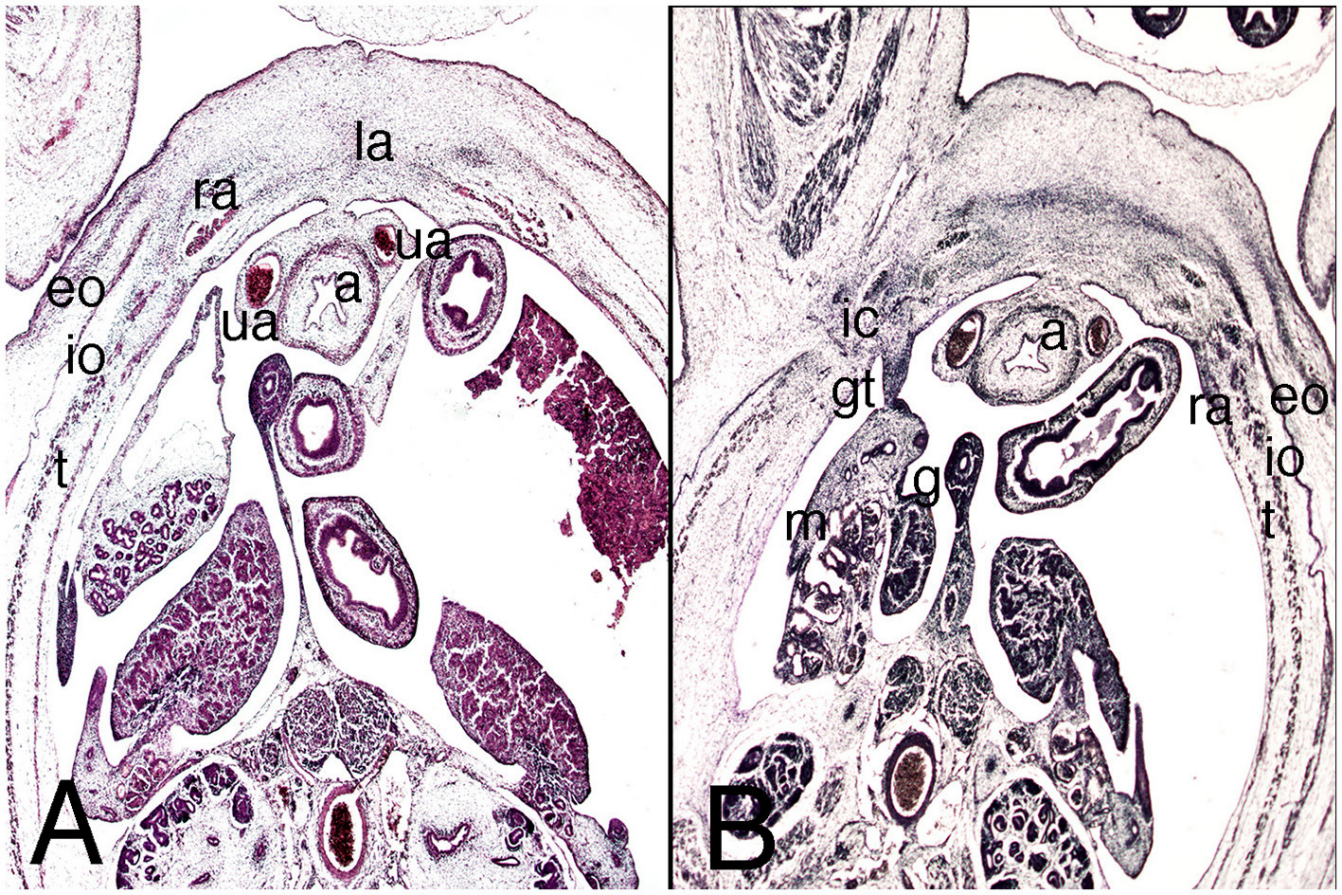
Cross sections of the abdominal cavity of the embryo MARC-1 (stage 21). Hematoxylin and eosin staining. **(A)** (2X) at the level of the infra-umbilical region, upper third; **(B)** (2X) at the lower third, inguinal canal. a, allantoid; eo,external oblique, g, gonad; gt, gubernaculum testis; ic, inguinal canal; io, internal oblique; la, linea alba; m, mesonephros; ra, rectus abdominis; t, transversus abdominis; ua, umbilical artery.

A small umbilical hernia persists in the abdominal wall, and the infra-umbilical region of the wall has enlarged cranially (Figure 6A). In contrast to previous stages, the three lateral abdominal muscles are clearly visible throughout their dorsoventral extension in the umbilical region, their aponeuroses surrounding the rectus abdominis (Figure 6B). This is now visible and large, situated in front of the anterior abdominal muscle in the infra-umbilical region (Figure 7).

The Camper's and Scarpa's fasciae are differentiated for the first time in the loose connective tissue of the superficial fascia or subcutaneous tissue (Figure 7A).

In the lower third of the inferior abdominal wall, the subcutaneous orifice of the inguinal canal in the external oblique is clearly visible (Figure 7B). The inguinal canal is straight and the preperitoneal and subcutaneous orifices are juxtaposed (Figure 7B).

For the first time, in relation to the inguinal portion, the parietal sheet of the peritoneum marks out a small recess called the vaginal process, which forms between the inguinal portion of the gubernaculum testis and the wall, marking out the preperitoneal orifice of the inguinal canal (Figure 7B).

### Fetuses of 9 Weeks

**Figure 8:**
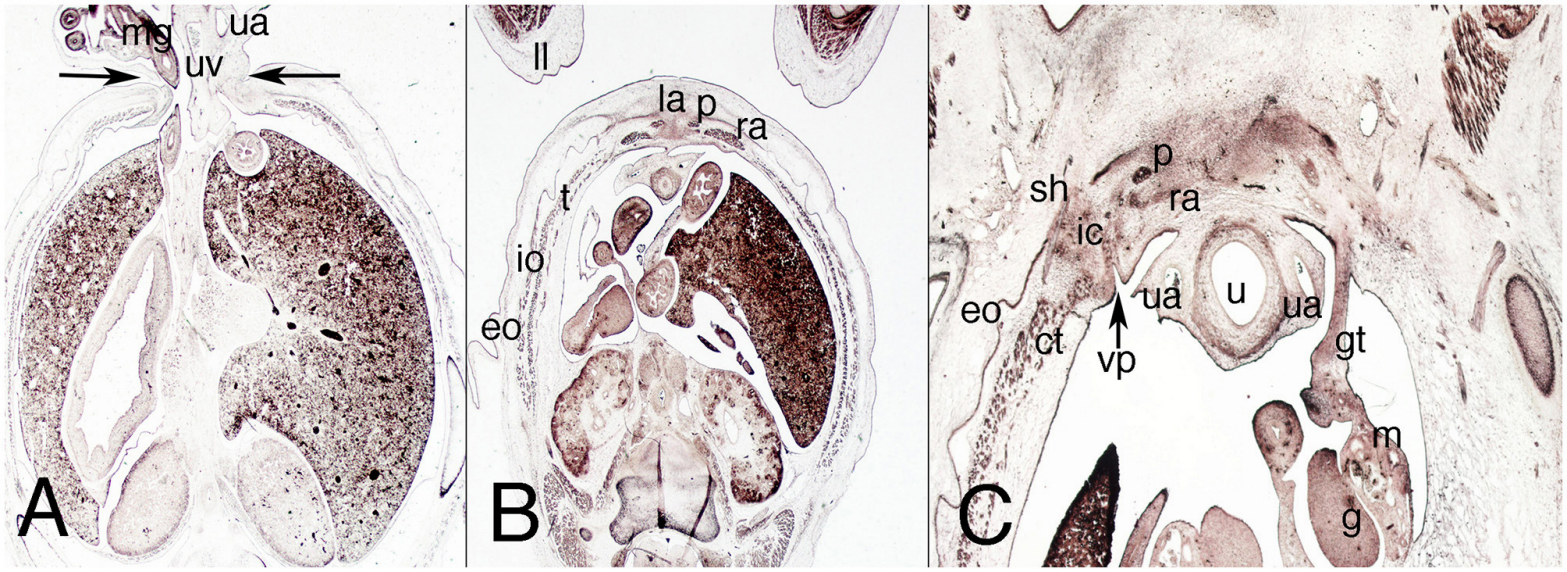
Transverse sections of the female fetus VD-34 (nine weeks) cranial to caudal. H-E, Azan and VOF staining. **(A)** (1x) level of the umbilical hernia; **(B)** (1x) section at middle third of the abdomen; **(C)** (1X) at the inferior third of the abdomen, inguinal region. ct, common tendon; eo, external oblique muscle; g, gonad; gt, gubernaculum testis; ic, inguinal canal; io, internal oblique muscle; la, linea alba; m, mesonephros; mg, middle gut; p, pyramidalis muscle; ra, rectus abdominis; sh, subcutaneous hole; u, urachu; ua, umbilical artery; uv, umbilical vein; vp, vaginal process. Arrows, neck of the umbilical hernia.

The physiological umbilical hernia in the abdominal wall shrinks, and the side of the infra-umbilical region expands (Figure 8A). The hernia is clearly encircled by the rectus abdominis muscle with its aponeurosis and the three layers of lateral abdominal muscles (Figures 8A,B). The pyramidalis muscle appears ventrally to the anterior abdominal muscle and is relatively bigger than in the adult (Figure 8B).

The inguinal canal is configured differently from the embryonic period because the preperitoneal and subcutaneous orifices have changed orientation and are now similar to the adult (Figure 8C). The anterior wall of the canal is formed by the aponeurosis of the external oblique muscle, the posterior wall comprises the transversalis fascia and Hesselbach's ligament, and the canal is bounded cranially by the common tendon and caudally by the inguinal ligament (Figure 8C). Inside the canal is the dense connective tissue of the gubernaculum testis (Figure 8C).

The nerves are readily observable in the nervous stratum, and the anterior scrotal branch of the ilioinguinal nerve exits the external orifice of the inguinal canal to distribute itself along the subcutaneous tissue of the future scrotal region (Figure 8C).

### Fetuses of 10 Weeks

**Figure 9:**
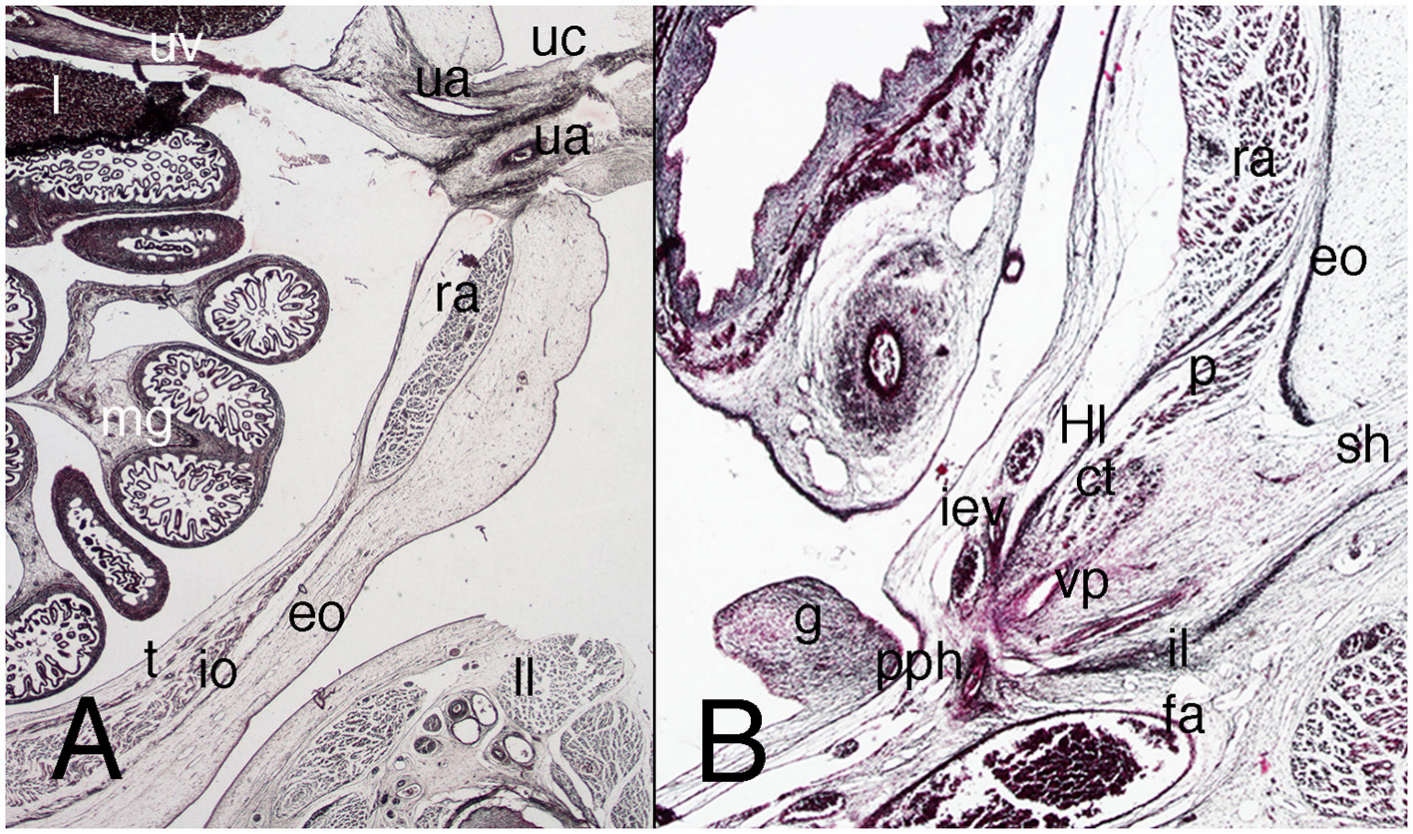
Sagittal sections of the abdomen in a female fetus F-25 (10th week). Azan staining. **(A)** (1X) at the umbilical and infra-umbilical regions; **(B)** (2X) section of the inguinal region. ct, common tendon; eo, external oblique muscle; fa, femoral artery; g, gonad; iev, inferior epigastric vessels; io, internal oblique muscle; Hl, Hesselbach's ligament; l, liver; mg, middle gut; p, pyramidalis muscle; pph, preperitoneal hole; ra, rectus abdominis; sh, subcutaneous hole; t, transversus muscle; ua, umbilical artery; uc, umbilical cord; uv, umbilical vein.

The umbilical hernia has become the umbilical cord (Figure 9A) and the inguinal canal shows the same orientation and morphological relationships as in 9-week-old fetuses. In the posterior wall of the transversalis fascia, it is much better differentiated than it was previously (Figure 9B). The lateral and anterior muscles of the abdomen are better defined, and the inguinal canal is clearly defined with its topographical relationships (Figure 9B).

### Fetuses of 11 and 12 Weeks

**Figure 10:**
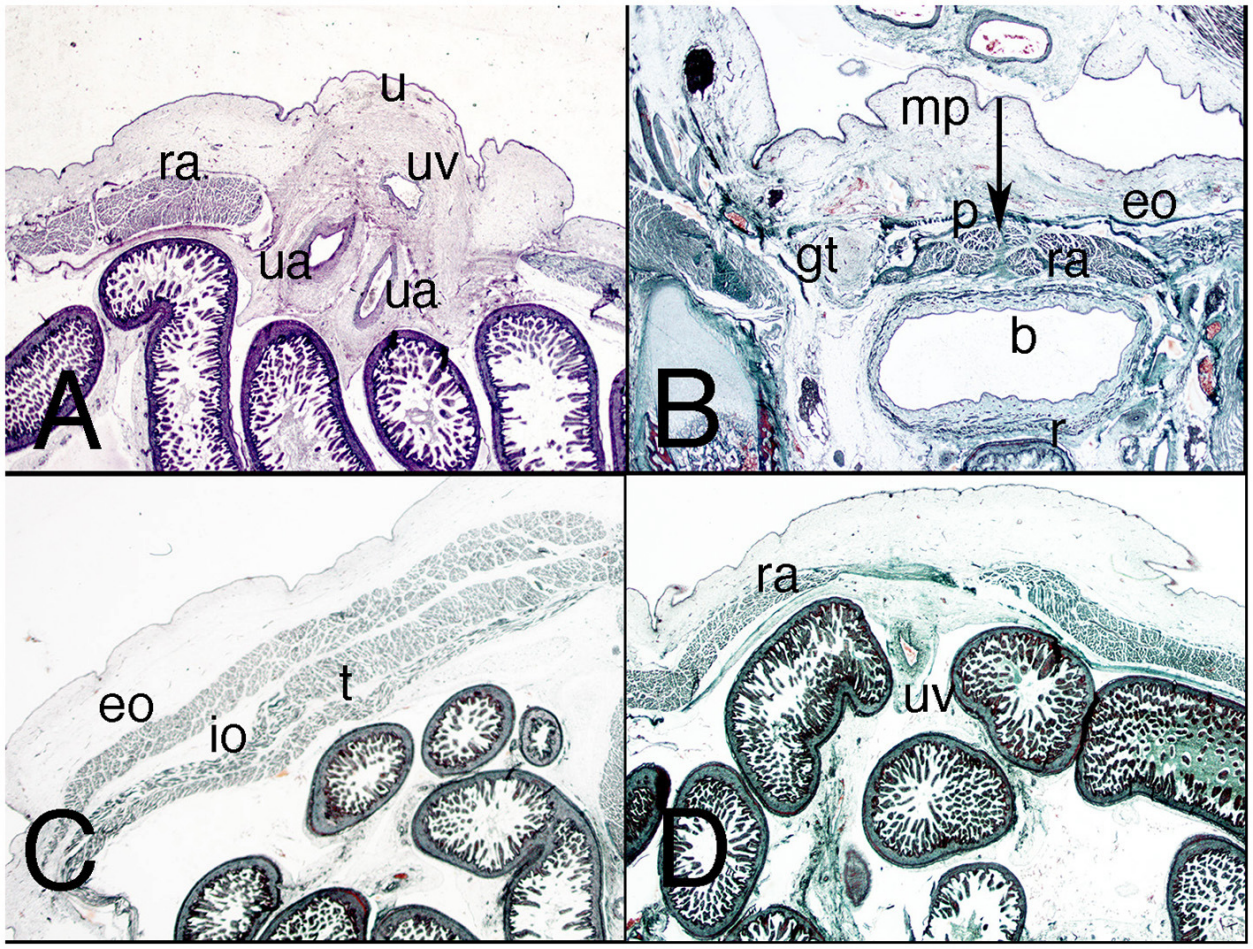
Axial sections of the abdomen in a fetus F-88 (12th week). Trichromic staining. **(A)** (1X) at the umbilicus region; **(B)** (2X) section of the inguinal region; **(C)** (1X) section of the middle third of the lateral abdominal wall; **(D)** (1X) section of the middle third of the anterior abdominal wall. b, bladder; eo, external oblique muscle; gt, gubernaculum testis; iev, inferior epigastric vessels; io, internal oblique muscle; mp, mons pubis; p, pyramidalis muscle; r, rectum; ra, rectus abdominis; t, transversus muscle; u, umbilicus; uv, umbilical vein. Arrow, linea alba.

The details on the abdominal wall are not much different from those in younger fetuses, but the umbilicus is clear (Figure 10A). The muscles have enlarged, and the topographical relationships have changed, their aponeuroses surrounding the rectus abdominis (Figures 10C,D). The anterior abdominal muscles sheath is formed by fibers from the three lateral muscles of the abdominal wall. The pyramidalis muscle is visible, more ventral than the anterior abdominal muscle and more proportionally sized in comparison to its adult dimensions (Figure 10B). The crossover of the rectus sheath defines a clear linea alba (Figure 10B).

The inguinal canal varies as the transversalis fascia of the posterior wall becomes denser (Figure 10B); the inguinal ligament is more evident, and the common tendon is thicker.

In men, the vaginal process surrounds the gubernaculum testis up to its inguinal portion; in a female fetus, it surrounds it only at the beginning of the initial course of the inguinal portion (Figure 10B).

### Fetuses of 14 and 15 Weeks

**Figure 11:**
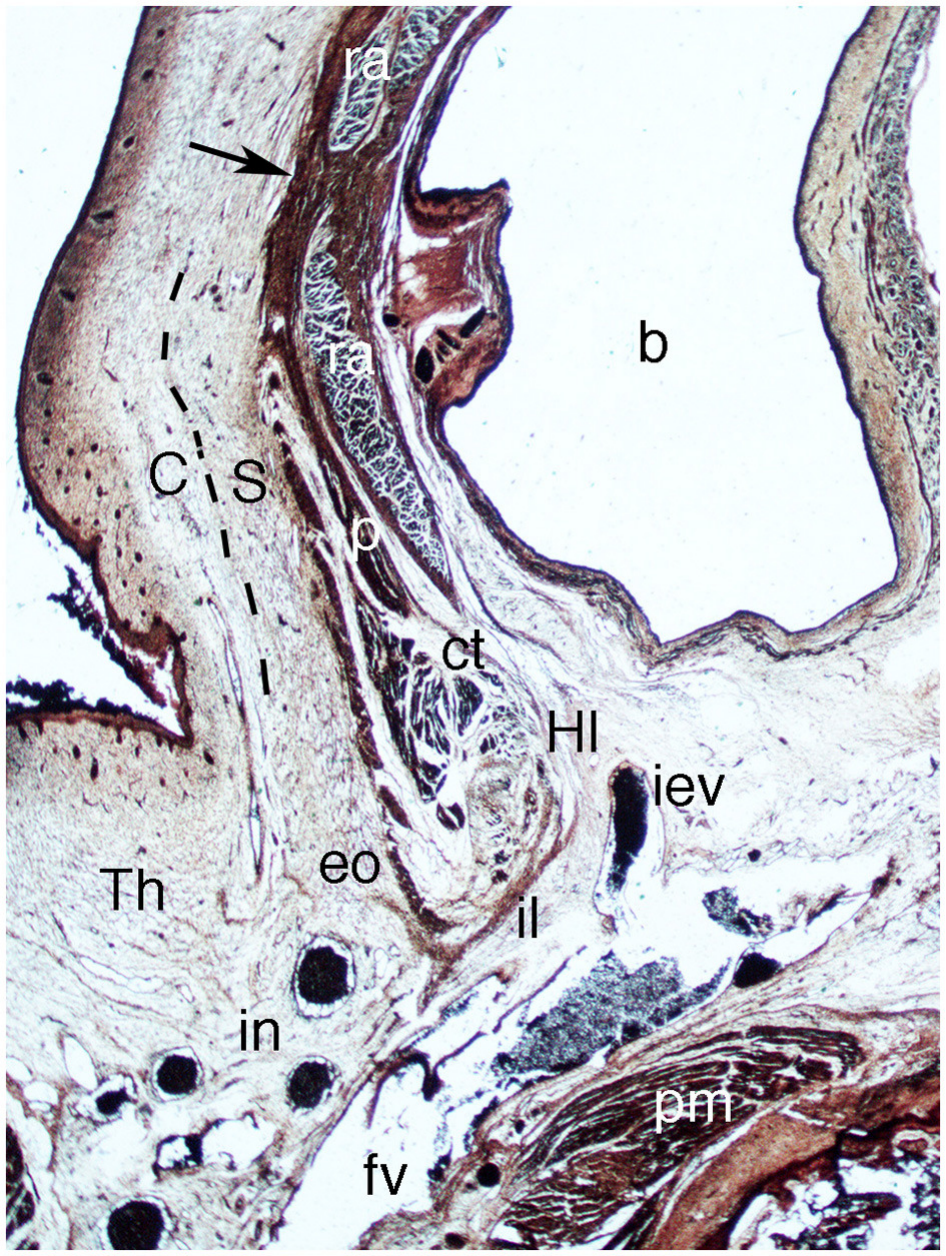
Sagittal section of the abdomen in fetus F-110 (15th week). Azan staining (1X). b, bladder; C, Camper's fascia; ct, common tendon; eo, external oblique muscle; fv, femoral vein; Hl, Hesselbach's ligament; iev, inferior epigastric vein; il, inguinal ligament; in, inguinal nodes; p, pyramidalis muscle; pm, pectineus muscle; ra, rectus abdominis; S, Scarpa's fascia; Th, thigh. Arrow, aponeurotic band.

In the abdominal wall, the pyramidalis muscle is more ventrally situated than the anterior abdominal muscle and is more proportionally sized in comparison to the adult. The walls and orifices of the inguinal canal are little changed from previous weeks, with clearly defined surfaces and topographical relationships (Figure 11). No vaginal process is visible in these two female fetuses (Figure 11). The Camper's and Scarpa's fasciae are differentiated very clearly in the loose subcutaneous cellular tissue (Figure 11).

## Discussion

The results will be discussed in three sections: abdominal wall and umbilical hernia, abdominal muscles, and inguinal canal. In general, they show that all elements appear at the end of the embryonic period proper and are clearly differentiated during the very early fetal period (9th−15th weeks).

### Abdominal Wall and Umbilical Hernia

The staging of secondary abdominal muscle development has never been described in detail in humans. In fact, there have been few studies, and these date only to the beginning of the 20th century (2–4). Recently, studies of embryos and fetuses using magnetic resonance microscopic and 3D reconstructions have been incorporated, but they focus on the development of the intestine and umbilical hernia not that of the muscular wall (6, 7).

The classical studies coincide with our results. However, these were based on studies of no more than three embryos, so our results are more precise. The primary loop of the intestine enters the umbilical cord during stages 14–16 (32–37 days; 6–10 mm), and during the rest of the embryonic period stage 23 (57 days; 37 mm) a big herniated umbilical sac occupies the umbilical hernia surrounded by the amniotic cavity (5–7, 20).

Similarly, all authors agree that during the early fetal period (9th−15th week) the intestine coils return to the abdominal cavity. This has been attributed to several factors: enlargement of umbilical arteries (2, 20), an increase of amniotic pressure, and a decrease in liver size (5). Interestingly, no author has considered the association of that reduction with the growth of the abdominal muscles.

However, as described by the classical authors, our results show that in stages 19 and 20 (48–51 days) the umbilical hernia starts to shrink and its neck narrows. During stages 21, 22, and 23 (51–57 days) up to the early fetal period (9th−10th week), the neck continues to narrow and the loops return to the abdominal cavity (2, 5).

Recently, a study of two embryos of stage 21 (52 days) with omphaloceles demonstrated that the myoblasts had migrated only 50–60% of the distance toward the ventral midline (9). Therefore, those authors consider that the omphalocele caused an arrest of the muscular migration at stage 19 (48 days) (9). Our results support that interpretation: the muscles of the anterior wall come to encircle the neck of the umbilical cord and the umbilicus is covered by the aponeurosis sheet.

### Abdominal Muscles

Few studies have analyzed the development of abdominal musculature, and most of them were published at the beginning of the 20th century (3, 4). Those classical studies used few specimens, four in total, with big gaps between stages. The authors consider that the abdominal musculature grows forwards from the somite (2–4). Our results and those of the classical authors show that in embryos of stage 15 (33 days) the abdominal musculature appears as a dorsal condensation of the mesenchyme or common myoblast mass at the dorsal level. During this period it is impossible to individualize any particular muscle. The mass continues ventrally by loose mesenchyme tissue up to the merger of the umbilical hernia and pelvis brim.

The next step could be observed in embryos of stage 16 (37 days; 9–14 mm): the abdominal muscles continued as a lateral sheet with two layers, internal and external. The external sheet represents the outlines of the external oblique muscle and the internal sheet the internal oblique and transversus abdominis muscles (3, 4).

In contrast to previous stages, stages 17 and 18 (41–44 days) witness differentiation of the musculature along a dorsal-ventral gradient; the muscle condensation proceeds ventrally. The internal oblique and transversus abdominis muscles end ventrally in a mass that represents the primordia of the rectus abdominis, which is connected to the neck of the umbilical hernia by loose connective tissue. During stages 19 and 20 (48–51 days) the outline of the three lateral abdominal muscles appears for the first time, the external oblique muscle being the best differentiated; but the internal oblique and transversus abdominis muscles are also differentiated, and anterior to them is the rectus abdominis.

Like those of earlier authors (2–4), our results show that during stages 21–23 (51–57 days), the end of the embryonic period, the abdominal muscles are differentiated and occupy the whole ventral and lateral abdominal wall encircling the future umbilical cord. During this period the aponeurosis crosses over the rectus abdominis and forms the rectus sheet and the linea alba. The pyramidalis muscle and the Camper's and Scarpa's fasciae in the subcutaneous tissue are also differentiated at this time. It is important to mention that throughout the development of the abdominal muscles, the ilioinguinal and iliohypogastric nerves appear as thick neural structures. This is consistent with the classical assumption that muscle development is linked to the presence of neural fibers (21).

Our results also confirm earlier findings that the muscles have finished differentiating, and their topographical relationships are established, during the early fetal period (9th−10th weeks). During the rest of the early fetal period they enlarge and mature and become more clearly defined (11th−15th weeks).

According to some authors the muscles do not fuse at the medial line until a later period, 3 months (6 cm) (3, 4). Therefore, the closing of the umbilical hernia coincides with the development of the abdominal muscles. We can consider that omphalocele could be explained by failure and delay in the differentiation and migration of myoblasts (9).

### Inguinal Canal

Felix in 1912 is categorical in affirming that the inguinal canal appears around an already formed gubernaculum testis and is initially developed with the external or subcutaneous orifice (22). Wyndham in 1943 and Backhouse in 1982 support this view (23, 24). Other studies mention that during its development the inguinal canal is occupied by the gubernaculum testis, which is covered by a vaginal tunic (13, 15, 16), and that its development is completed right before the end of the embryonic period; in the eighth week (16), as we also found; or in the 20th week of fetal development (15), which is not consistent with our findings. None of the authors considered in this study mention the orientation and dimensions of the inguinal canal. We ascertained that at the end of the embryonic period the canal is short and straight with its two orifices juxtaposed. This relationship changes at the beginning of the fetal period, between the 9th and 12th week, when the subcutaneous orifice becomes more medial and the preperitoneal orifice more lateral, as in adult life.

## Data Availability Statement

The original contributions presented in the study are included in the article/supplementary material, further inquiries can be directed to the corresponding author.

## Ethics Statement

Ethical review and approval was not required for the study on human participants in accordance with the local legislation and institutional requirements. The patients/participants provided their written informed consent to participate in this study.

## Author Contributions

JB, CG, MK, and JS contributed to conception and design of the study. JB, CG, and JS wrote the first draft of the manuscript. MK, RT, and EP wrote sections of the manuscript, gave content for analysis and interpretation for the work, and revising it critically for important intellectual content. All authors contributed to manuscript revision, read, and approved the submitted version.

## Conflict of Interest

The authors declare that the research was conducted in the absence of any commercial or financial relationships that could be construed as a potential conflict of interest.

## Publisher's Note

All claims expressed in this article are solely those of the authors and do not necessarily represent those of their affiliated organizations, or those of the publisher, the editors and the reviewers. Any product that may be evaluated in this article, or claim that may be made by its manufacturer, is not guaranteed or endorsed by the publisher.

## References

[1] Bouzada J Vazquez T Duran M Delmas V Larkin T Cuesta MA. New insights into the morphogenesis of the gubernaculum testis and the inguinal canal. Clin Anat. (2017) 30:599–607. 10.1002/ca.22880 28422355

[2] Mall FP. Walls in man. J Morphol. (1898) 14:347. 10.1002/jmor.1050140208

[3] Bardeen CR Lewis WH. Development of the limbs, body-wall and back in man. Am J Anat. (1901) 1:1–35. 10.1002/aja.1000010102

[4] Lewis WH. The Development of the Muscular System. Manual of Human Embryology (1910).

[5] Frazer JE Robbins RH. On the factors concerned in causing rotation of the intestine in man. J Anat Physiol. (1915) 50:75–110. 17233053 PMC1289077

[6] Ueda Y Yamada S Uwabe C Kose K Takakuwa T. Intestinal rotation and physiological umbilical herniation during the embryonic period. Anat Rec. (2016) 299:197–206. 10.1002/ar.23296 26599074

[7] Nagata A Hatta S Ji X Ishikawa A Sakamoto R Yamada S. Return of the intestinal loop to the abdominal coelom after physiological umbilical herniation in the early fetal period. J Anat. (2019) 234:456–64. 10.1111/joa.12940 30681143 PMC6422813

[8] Ji X Ishikawa A Nagata A Yamada S Imai H Matsuda T. Relationship between rectal abdominis muscle position and physiological umbilical herniation and return: a morphological and morphometric study. Anat Rec Adv Integr Anat Evol Biol. (2020) 303:3044–51. 10.1002/ar.24358 31908135

[9] Nichol PF Corliss RF Yamada S Shiota K Saijoh Y. Muscle patterning in mouse and human abdominal wall development and omphalocele specimens of humans. Anat Rec. (2012) 295:2129–40. 10.1002/ar.22556 22976993 PMC3976953

[10] Eccles WM. The Imperfectly Descended Testis: Its Anatomy, Physiology and Pathology. Wood (1903).

[11] Keith A. On the origin and nature of hernia. Br J Surg. (1924) 11:455–75. 10.1002/bjs.1800114307

[12] Tillaux PJ Y Sabater JC Pérez AM. Tratado de Anatomía Topográfica Aplicada a la Cirugía. Biblioteca Ilustrasda de Espasa y Compañía (1880).

[13] Hart DB. The nature and cause of the physiological descent of the testes. J Anat Physiol. (1909) 44:4. 17232824 PMC1289220

[14] Hart DB. The physiological descent of the ovaries in the human foetus. J Anat Physiol. Edinburgh: Royal College of Physicians (1909) 44:27–34. 17232822 PMC1289221

[15] Barteczko KJ Jacob MI. The testicular descent in human. Origin, development and fate of the gubernaculum Hunteri, processus vaginalis peritonei, gonadal ligaments. Adv Anat Embryol Cell Biol. (2000) 156:III–X:1–98. 10.1007/978-3-642-58353-7 11008363

[16] Niikura H Okamoto S Nagase S Takano T Murakami G Tatsumi H. Fetal development of the human gubernaculum with special reference to the fasciae and muscles around it. Clin Anat. (2008) 21:547–57. 10.1002/ca.20675 18661576

[17] O'rahilly R. Developmental stages in human embryos including a revision of Streeter's “horizons” and a survey of the Carnegie Collection. Contrib Embryol Carneg Inst. (1987) 637:65–201.

[18] Sanudo JR Domenech-Mateu JM. The laryngeal primordium and epithelial lamina. A new interpretation. J Anat. (1990) 171:207–22. 2081706 PMC1257142

[19] Patten. Embriologia Humana. 5th ed. (1973). p. 149–52.

[20] Lewis F. The Development of the Intestinal Tract and Respiratory Organs. Manual of Human Embryology. Philadelphia, PA: JB Lippincott Co. (1912). 302 p.

[21] Lexell J Sjostrom M Nordlund AS Taylor CC. Growth and development of human muscle: a quantitative morphological study of whole vastus lateralis from childhood to adult age. Muscle Nerve. (1992) 15:404–9. 10.1002/mus.880150323 1557091

[22] Felix W. The Development of the Urinogenital Organs. I. The Development of the Excretory Glands and Their Ducts. Manual of Human Embryology II. Philadelphia, PA: JB, Lippincott Co. (1912). p. 752–880.

[23] Wyndham NR. A morphological study of testicular descent. J Anat. (1943) 77:179–88. 17104926 PMC1252756

[24] Backhouse KM Butler H. The gubernaculum testis of the pig (Sus scropha). J Anat. (1960) 94:107–20. 13795600 PMC1244420

